# Lamivudine induced pure red cell aplasia and HIV-1 drug resistance-associated mutations: a case report

**DOI:** 10.1093/omcr/omad022

**Published:** 2023-03-25

**Authors:** Mireille A M Kakubu, Tarisai Bikinesi, Patrick D M C Katoto

**Affiliations:** Ministry of Health and Social Services of Namibia, Windhoek, Namibia; Ministry of Health and Social Services of Namibia, Windhoek, Namibia; Centre for Tropical Disease and Global Health, School of Medicine, Catholic University of Bukavu, Bukavu, Democratic Republic of Congo; Cochrane South Africa, South African Medical Research Council, Francie Van Zijl Drive, Parow Valley, 7501 Cape Town, South Africa

## Abstract

Adverse effects linked to antiretroviral therapy (ART) may contribute to poor adherence on the patient’s side. Consequently, human immunodeficiency virus (HIV) drug resistance mutations could emerge, negatively impacting the body’s immune system. Meanwhile, severe immunosuppression can lead to several conditions, including anemia. The cause of anemia in HIV infection is multifactorial, and can be mainly explained by deleterious direct effects of the virus on the bone marrow, and opportunistic infections such as Parvovirus B19. Other causes include blood loss resulting from neoplasms and gastrointestinal lesions. Moreover, anemia can also be caused by antiretroviral drugs. We report a case of persistent anemia after ART initiation, kidney injury and treatment failure following a lengthy period of non-adherence to ART. The anemia was classified as Pure Red Cell Aplasia (PRCA). With treatment modification, the anemia resolved and the patient attained virologic suppression. Lamivudine (3TC) was pointed out as the cause of PRCA, which resolved after its withdrawal from the ART regimen. This rare side effect should be investigated in patients on 3TC who present with recurrent anemia.

## INTRODUCTION

Anemia in people living with human immunodeficiency virus (PLHIV) has been associated with poor HIV treatment responses, high viral loads (VL), low CD4 counts and increased risk of death [1, 2]. Several medicines have been implicated in developing adverse drug events, compromising the quality of life [3]. Thus, patients lose interest by stopping adhering to antiretroviral therapy (ART), resulting in poor treatment outcomes [3].

Besides, Nucleoside Reverse Transcriptase Inhibitors (NRTIs) such as zidovudine (AZT) and lamivudine (3TC) have the propensity to cause hematologic toxicity, including anemia [4].

We present a patient assessed with severe persistent anemia, multiple treatment interruptions, HIV drug resistance and treatment failure months after ART initiation.

## CASE REPORT

This is a 44-year-old single and self-employed female who was initiated on tenofovir disoproxil fumarate (TDF), 3TC and efavirenz (EFV) as an outpatient in July 2012. Her baseline laboratory test results were as follows: CD4 count: 190 cells/mm^3^; hemoglobin (Hb): 10.8 g/dl; creatinine clearance: 63 ml/min and hepatitis B surface antigen (HBsAg): non-reactive. She neither had a history of alcohol intake nor cigarette smoking. She interrupted her ART 1 month after ART initiation. All efforts to retain her in care were unsuccessful.

Six years later, when she returned to care, she was not taking ART. She complained of chronic fatigue and was assessed with advanced HIV disease and kidney dysfunction. She was reinitiated on the same ART regimen (TDF + 3TC + EFV). At re-initiation of ART, her CD4 count was 33 cells/mm^3^, and her Hb was 9.6 g/dl. A gradual decline in her Hb level—from 9.6 g/dl to 6.3 g/dl—was observed over 19 months (February 2018 to September 2019). She neither had leucopenia nor thrombocytopenia; her CD4 count had dropped to 11 cells/mm^3^, and blood loss was excluded during this period. From February 2019 to January 2020, the HIV plasma viral loads were persistent at >1000 copies/ml despite sessions of enhanced adherence counseling. Her creatinine clearance dropped to 30 ml/min and TDF doses were adjusted accordingly.

**Table 1:** Mutations detected at the HIV-1 GRT, corresponding penalty scores and drug susceptibilities following analysis with the Stanford HIV drug resistance database version 9.0

ARVs class	Detected mutations	Penalty scores		Drug susceptibility
NRTIs	K70KE, M184V,	AZT	−20	Susceptible
		TDF	15	Low-level resistance
		ABC	30	Intermediate resistance
		3TC/FTC	70	High-level resistance
Non-NRTIs	V106VA, E138G, V179D, G190GA and P225H	ETR	30	Intermediate resistance
		NVP EFV	185 155	High-level resistance
Protease Inhibitors (PI) majors, accessories and other PI mutations	None	LPV/r	0	Susceptible
		ATV/r	0	Susceptible
		DRV/r	0	Susceptible

**Figure 1:**
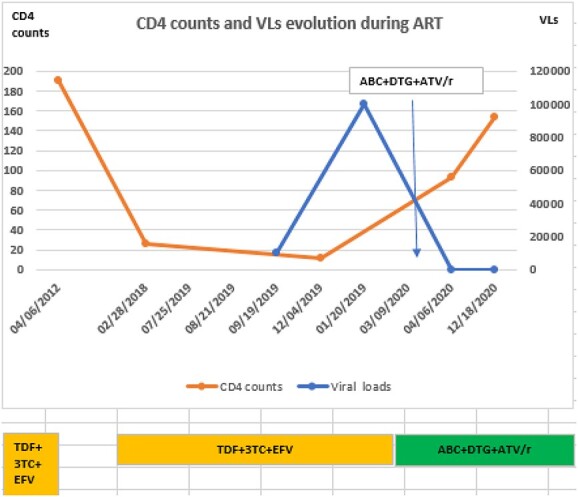
CD4 counts (cells/mm^3^) and VLs (copies/ml of plasma) evolution: A considerable drop in CD4 cell count and increase in VLs during the non-adherence time interval with the patient’s initial regimen (TDF + 3TC + EFV) was observed. Based on the findings, the initial regimen was switched to ABC + DTG + ATV/r, followed by improved immunologic status and virologic outcomes, as illustrated.

In July 2019, a full blood count showed normocytic, normochromic anemia and reticulocytes <1%. Additionally, bone marrow aspirate analysis revealed histologic features suggestive of Pure Red Cell Aplasia (PRCA), whereas, polymerase chain reactions (PCR) on the bone marrow biopsy were negative for Parvovirus B19, Cytomegalovirus, Epstein Bar Virus and mycobacterium avium complex. The protein electrophoresis and immunofixation were negative for multiple myeloma. The HBsAg remained non-reactive, as well as the cryptococcal serum antigen and tuberculosis screenings, and the creatinine clearance was estimated at 36 ml/min.

The patient was initiated empirically on steroids in the oncology department for 7 months to control possible immunologic disorders commonly associated with anemia in PLHIV. During the course of the treatment, her Hb slightly improved to 8.5 g/dl. However, during the same period, she suffered from esophageal candidiasis, an acquired immunodeficiency syndrome (AIDS)-defining condition treated successfully with fluconazole.

In January 2020, she was referred to an HIV specialist who diagnosed her with HIV treatment failure and PRCA. Hence, 3TC was suspected to be the cause of PRCA. The steroids were discontinued, and the Sanger genotyping resistance testing (GRT) exposed these mutations: K70KE, M184V, V106VA, E138G, V179D, G190GA and P225H in the reverse transcriptase enzyme, but no mutations in the viral protease enzyme (Table 1). The HIV Drug Resistance Database of Stanford University version 9.0 was used to predict drug activity [5]. The genotyping results and drug toxicity profiles were used to guide the optimization of the ARV regimen.

**Figure 2:**
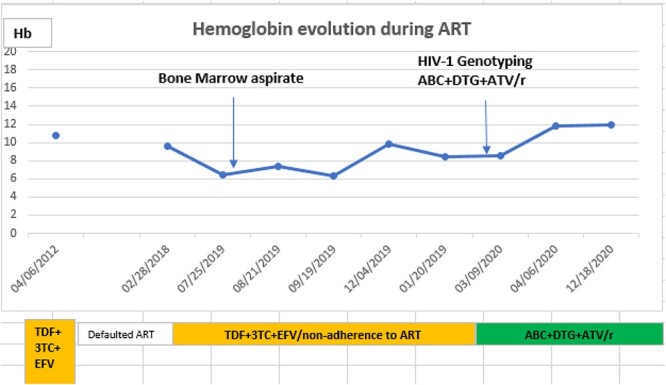
Hemoglobin (g/dl)' profile during ART: The initial patient’s Hb levels persistently below 10 g/dl while on 3TC containing ART regimen improved on ABC + DTG + ATV/r.

End of February 2020, 3TC was withdrawn from the regimen, and a combination of drugs that pharmacokinetics does not affect kidney function was initiated; abacavir (ABC) + atazanavir/ritonavir (ATV/r) + dolutegravir (DTG). Over a period of 9 months, the patient’s (Fig. 2) creatinine clearance improved to >60 ml/min; her CD4 count increased to 154 cells/mm^3^, her plasma VL was maintained at <40 copies/ml of plasma (Fig. 1) and the Hb rose to >11.5g/dl (Fig. 2).

## DISCUSSION

Although anemia is frequently seen in PLHIV, rare side effects of ARVs should be investigated and managed accordingly. HIV disease severity is one of the predisposing factors to anemia [5], and anemia increases mortality and morbidity in PLHIV [6]. The above patient-initiated ART in a state of severe immunosuppression with a CD4 count <200 cells/mm^3^. Her low baseline Hb was expected to be corrected after ART initiation and good adherence [7]. However, 1 month later, the patient discontinued ART for ˃5 years.

Bukenya *et al*. reported that fear of ART-related side effects was associated with non-adherence and treatment failure [8]. A decline in the patient’s general condition was believed to be attributed to ART side effects that led to treatment interruption. After a lengthy period off ART, the patient returned to care with a very low CD4 count.

ART was re-started with the initial regimen namely—TDF, 3TC and EFV. A combination of AZT and 3TC can potentially cause PRCA, a rare hematologic adverse event [4]. This line of thought led the clinicians who managed the patient to have a low index of suspicion toward 3TC as a single cause of anemia, despite its recurrence and histologic findings on the bone marrow biopsy. Besides, the slight improvement in Hb level while on steroids contributed to misleading the clinician. However, Nakamura *et al*. [9] argued that 3TC in the absence of AZT could induce anemia.

Only 7 months after treatment with steroids, an HIV-experienced clinician, guided by the patient’s medical history, suspected 3TC as the culprit. The PRCA could have resulted from HIV, multiple myeloma or opportunistic infections such as parvovirus B19 [10, 11]. However, the negative PCR results for opportunistic infections and protein electrophorese for multiple myeloma contributed to the diagnosis of the 3TC-induced PRCA.

Regarding renal function, a decline in creatinine clearance was observed in the same period—following ART re-initiation. TDF causes proximal tubular cell injury, which aggravates pre-existent kidney diseases, such as HIV-associated Nephropathy, a condition frequently observed in patients of African descent [12]. As such, TDF was substituted with ABC followed by improved creatinine clearance.

Overall, 3TC remained this patient’s sole cause of anemia, and its withdrawal from the ART regimen was corroborated by improved patient’s general condition and raised Hb levels. However, symptomatic anemia associated with 3TC was responsible for non-adherence, resulting in extensive HIV-1 reverse transcriptase mutations and treatment failure, which was addressed with a treatment switch.

## CONCLUSION

Delayed work-up of the persistent symptomatic anemia negatively impacted the patient’s adherence to ART, and treatment failure ensued. We want to draw the reader’s attention to the rare and reversible drug toxicity profile of 3TC since earlier detection could have prevented non-adherence and the selection of mutations in the HIV-1 reverse transcriptase.

## Supplementary Material

3TC_Anemia_HIV_DR_cover_letter_omad022
