# Supplementary material for: Lamivudine induced pure red cell aplasia and HIV-1 drug resistance-associated mutations: a case report
Source: Oxf Med Case Reports. 2023 Mar 25;2023(3):omad022. doi: 10.1093/omcr/omad022 (PMC10041944; doi:10.1093/omcr/omad022)
Supplement: 3TC_Anemia_HIV_DR_cover_letter_omad022 [file 3tc_anemia_hiv_dr_cover_letter_omad022.docx]

Mireille A Mpalang Kakubu

Regional HIV Clinical Mentor

Ministry of Health of Namibia

Windhoek

Namibia

05/08/2022

Dear Editor,

On behalf of the other authors, we want to publish the manuscript of the case study entitled " **A case report of Lamivudine Induced Pure Red Cell Aplasia and HIV-1 drug resistance.**" for consideration by Oxford Medical Case Reports Journal.

This case report is an original work, accepted as an oral presentation at the 29th International Workshop of HIV Drug Resistance and Treatment strategies held in September 2021. This work has not been submitted for publication elsewhere.

ART side effects can impact the treatment outcome. We present a case of a patient who presented with severe persistent anaemia rarely caused by Lamivudine. The diagnosis was made late during the treatment and caused poor patient adherence, HIV resistance and therapy failure months after ART initiation.

We believe that this case report fits the aims and scope of the Journal of International Association of Providers of AIDS Care, will interest the readers and will contribute to improving the management of HIV-infected patients.

We have no conflict of interest.

Thank you for your consideration of this case report.

Sincerely,

Mireille A Mpalang Kakubu

[mpalangkakubu@gmail.com](mailto:mpalangkakubu@gmail.com)
